# Magnetic Resonance Imaging as a Biomarker in Rodent Peripheral Nerve Injury Models Reveals an Age-Related Impairment of Nerve Regeneration

**DOI:** 10.1038/s41598-019-49850-2

**Published:** 2019-09-18

**Authors:** Elisa Giorgetti, Michael Obrecht, Marie Ronco, Moh Panesar, Christian Lambert, Nathalie Accart, Arno Doelemeyer, Mark Nash, Michael Bidinosti, Nicolau Beckmann

**Affiliations:** Novartis Institutes for BioMedical Research, Musculoskeletal Diseases Department, CH-4056 Basel, Switzerland

**Keywords:** Diagnostic markers, Peripheral nervous system

## Abstract

Assessment of myelin integrity in peripheral nerve injuries and pathologies has largely been limited to post-*mortem* analysis owing to the difficulty in obtaining biopsies without affecting nerve function. This is further encumbered  by the small size of the tissue and its location. Therefore, the development of robust, non-invasive methods is highly attractive. In this study, we used magnetic resonance imaging (MRI) techniques, including magnetization transfer ratio (MTR), to longitudinally and non-invasively characterize both the sciatic nerve crush and lysolecithin (LCP) demyelination models of peripheral nerve injury in rodents. Electrophysiological, gene expression and histological assessments complemented the extensive MRI analyses in young and aged animals. In the nerve crush model, MTR analysis indicated a slower recovery in regions distal to the site of injury in aged animals, as well as incomplete recovery at six weeks post-crush when analyzing across the entire nerve surface. Similar regional impairments were also found in the LCP demyelination model. This research underlines the power of MTR for the study of peripheral nerve injury in small tissues such as the sciatic nerve of rodents and contributes new knowledge to the effect of aging on recovery after injury. A particular advantage of the approach is the translational potential to human neuropathies.

## Introduction

Myelin content is commonly used as an indicator of nerve health since myelin reduction is often associated with neural pathology such as multiple sclerosis (MS), trauma and dementia (see^1^ for a review), as well as with normal aging^2^. The term demyelination describes the damage or loss of healthy myelin around axons in diseases characterized by injury to the myelin sheaths. Dysmyelination can also occur as a result of genetic or acquired defects in the cells that form them (oligodendrocytes in the central nervous system and Schwann cells in the peripheral nervous system), with relative sparing of axonal damage.

Diagnosis of demyelination or dysmyelination carries important therapeutic and prognostic implications. Electromyography and nerve conduction studies are routinely used to assess peripheral nerves lesions^3,4^. However, they are not characteristic of a specific disease or able to give a definitive diagnosis, and have some limitations that may lead to misdiagnosis. Age of the patient, body temperature as well as length of limbs and nerve are some of the factors that may affect outcome^5^. Histopathology is largely confined to post-*mortem* confirmation and only occasionally included for accurate diagnosis of the presence or cause of impaired myelin integrity before death. Since the 1990s, magnetic resonance imaging (MRI) has shown increasing value in this area both preclinically and clinically. MRI techniques such as diffusion-weighted imaging (DWI), diffusion tensor imaging (DTI), and magnetization transfer ratio (MTR) imaging have been applied to the study of axons. DWI provides information on the structural integrity of nerve white matter by measuring the local diffusion characteristics of water, which is influenced by the integrity of myelin and axonal membranes. In contrast to the non-directional measure of diffusion given by DWI, DTI provides directional information on water diffusion. Similarly to diffusion MRI, MTR imaging is sensitive to changes in myelin density that can occur in neuropathy^6,7^.

Most studies involving the use of MRI to examine axonal pathology were devoted to the central nervous system (see^8–11^ for reviews). However, MRI has also been used to assess axonal pathology in the periphery. MR neurography leveraging fast spin echo and diffusion-weighted imaging techniques to provide high-resolution, nerve-selective images has become an important tool for the precise spatial detection of lesions in focal and non-focal disorders of the peripheral nervous system^12–15^. Axonal nerve injury leads to Wallerian degeneration, resulting in a hyper-intense nerve signal on T_2_-weighted MR images of the degenerating distal nerve segment^16^. Various combinations of non-specific tissue alterations, such as inflammation, demyelination or axonal injury, can cause these signal changes^17^. Contrast agents, including gadofluorine M and superparamagnetic iron oxide particles, allow the visualization of both the dynamics of peripheral nerve injury and its repair^18,19^ and of macrophage infiltration^20^, respectively. Both have been used to increase specificity in experimental models. Although DTI enables the assessment of nerve repair^21–23^, its preclinical use has so far been limited to analyses of excised nerves that last several hours^24^. This indicates the challenge of incorporating this technique into routine preclinical studies with small rodents. However, as an alternative, Dortch *et al*.^25^ introduced the magnetization transfer ratio (MTR) as a potential biomarker of proximal sciatic nerve dysmyelination pathology in patients with Charcot-Marie-Tooth (CMT) disease.

MTR imaging has been used to non-invasively assess demyelination processes in the mouse brain^26–29^ and spinal cord^30^. Here, we examined the feasibility of adapting it to investigate peripheral nerve injury and repair in small rodent models of acute sciatic nerve injury. The broader objective was to understand the translation potential for MTR as a diagnostic and pharmacodynamic biomarker for the development of therapies aimed at accelerating peripheral nerve repair after injury. In this study, MTR of the sciatic nerve was complemented by *in vivo* electrophysiology and toe spread assessments, as well as by post-*mortem* histology and gene expression analyses in a murine sciatic nerve crush (SNC) model. These were correlated with the corresponding changes in muscle. To investigate the potential effect of age on the recovery of neuromuscular function, SNC was performed in young and aged wild type mice (9 weeks and 18 months at crush, respectively). A second model of demyelination by local injection of lysolecithin (LCP) in rat sciatic nerve was included for comparison.

## Results

### Effect of sciatic nerve crush (SNC) on magnetization transfer ratio (MTR)

**Figure 1:**
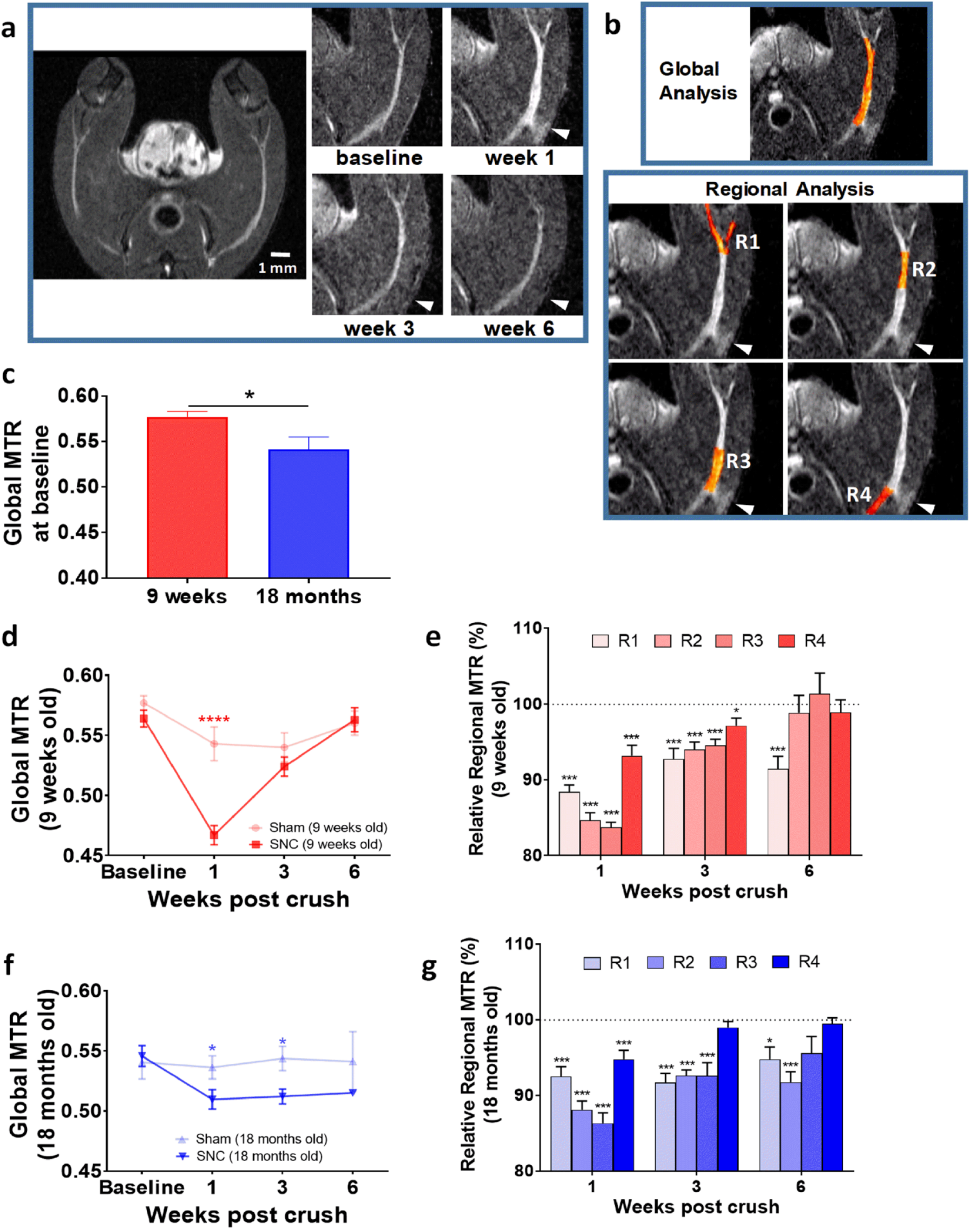
Age-dependent effects of SNC on MTR in mice. **(a)** Representative T_2_-weighted images at baseline and at week 1, 3 and 6 after SNC. Arrowhead indicates the site of crush. **(b)** Choice of regions-of-interest for a global and regional MTR analysis, with segments R1-4 placed in relation to the SNC location (arrowhead). **(c)** Global MTR determined at baseline in young and old mice (Means ± SEM; *0.01 < p < 0.05). **(d)** Global MTR in young mice (Means ± SEM; ****p < 0.0001, comparison sham vs SNC). **(e)** Regional MTR at week 1, 3 and 6 after SNC in young mice and expressed as relative to baseline values for each region (Means ± SEM; *0.01 < p < 0.05, ***p < 0.001, comparisons to baseline for each region). **(f)** Global MTR in old mice (Means ± SEM; *p < 0.05, comparisons sham vs SNC). **(g)** Regional MTR at week 1, 3 and 6 after SNC in old mice and expressed as relative to baseline values for each region (Means ± SEM; *0.01 < p < 0.05, ***p < 0.001, comparisons to baseline for each region). Number of mice in each age group: n = 12 sham and n = 18 SNC at baseline and week 1, n = 8 sham and n = 12 SNC at week 3, n = 4 sham and n = 6 SNC at week 6.

In this study we used MTR to assess nerve injury and recovery following SNC. Figure 1a shows typical T_2_-weighted MRI images from the mouse lower limbs, displaying the sciatic nerve and how it changes compared to baseline at week 1, 3 and 6 following SNC. Injured nerves exhibited marked enlargement and hyperintense signal at week 1 postoperatively. Such images were used for defining the regions-of-interest (ROIs) for MTR analyses. When applying MTR for the study of the sciatic nerve recovery after injury, the nerve was analyzed as a whole (global MTR) or by sub-dividing it in 4 regions, named R1 (most distal, at and after the branching point into tibial and peroneal nerves) to R4 (most proximal region to the lumbar vertebrae L4) (Fig. 1b). The nerve was crushed approximatively at the interface of regions R3 and R4.

Global MTR in old mice was significantly lower at baseline compared to young animals (Fig. 1c). At week 1 after SNC, global MTR in young mice dropped by 18.5% from approximately 0.57 to 0.47 and gradually recovered by week 3 (Fig. 1d). The sham procedure also influenced the nerve, since a small but not significant drop in global MTR was detected at week 1. Regional analysis revealed significant differences between MTR in proximal and distal regions at baseline (Fig. 1e and Suppl. Fig. 1a). Interestingly, a significant decline in MTR was observed as early as one week after SNC in all analyzed regions (R1-R4). At week 6, MTR in the three proximal regions to the crush (R2-R4) was equivalent to pre-surgery values, while MTR in distal region R1 was still significantly decreased.

With lower levels at baseline, global MTR in old mice following SNC declined to a lower degree than in the young cohort (Fig. 1f). Interestingly, despite displaying a less pronounced reduction, global MTR remained low throughout the experiment, when compared to sham-operated controls. MTR on sham-operated control hind limbs did not change at any time point. Regional analysis demonstrated significant differences between MTR in proximal and distal regions of the nerve (Fig. 1g and Suppl. Fig. 1b) in old animals during the recovery phase: in contrast to the observations made in young mice, MTR in distal regions (R1-R2) remained significantly low until week 6. A trend towards lower MTR compared to baseline persisted also in the proximal region R3 at week 6 (Fig. 1g). The MTR data suggest that the non-recovered area of the nerve at week 6 after SNC was larger in old as compared to young mice.

### Toe spread reflex and electrophysiological studies demonstrated a delayed functional recovery in old compared to young mice following SNC

**Figure 2:**
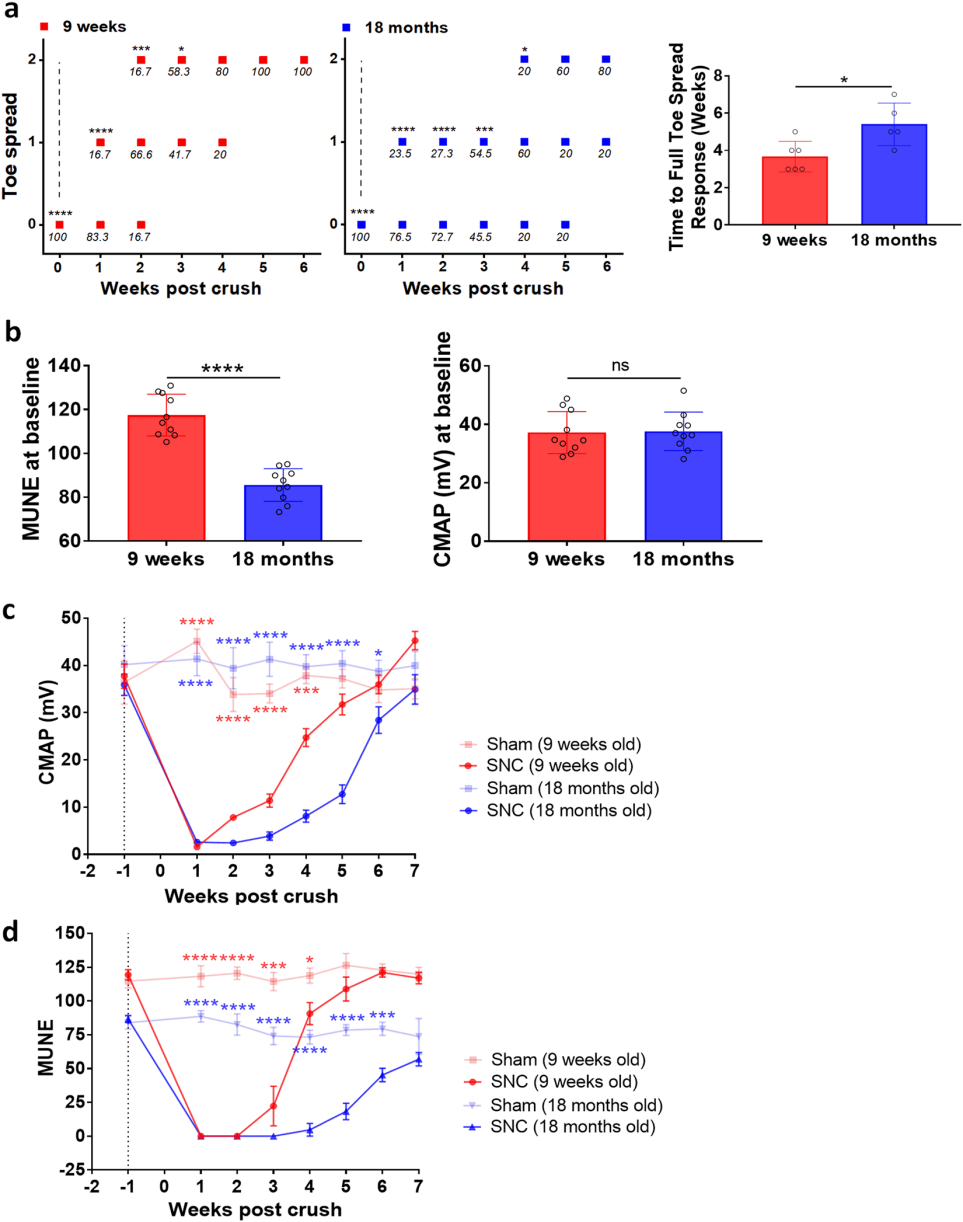
Delayed functional recovery in old compared to young mice following SNC. **(a)** Toe spread scores (0 - no spreading, 1 - intermediate spreading, 2 - full toe spreading) assessed weekly. The numbers in italics below the symbols indicate the percentage of animals receiving the score at that time point. The levels of significance *0.01 < p < 0.05, ***0.0001 < p < 0.001, ****p < 0.0001 correspond to comparisons to sham animals, that had scores of 2 at all time points. Data are also expressed as the time (means ± SEM) necessary to obtain a full toe spread response (score = 2). **(b)** EMG recordings performed at baseline (1 week before the SNC) to compare young and old mice. Data are expressed as means ± SEM; ****p < 0.0001, ns = not significant. **(c)** CMAP and **(d)** MUNE time course in SNC- and sham-operated mice (Means ± SEM; *0.01 < p < 0.05, ***0.0001 < p < 0.001, ****p < 0.0001, comparisons sham and SNC for each age group). Dotted line corresponds to baseline. Number of mice in each age group: n = 12 sham and n = 18 SNC at baseline and week 1, n = 8 sham and n = 12 SNC at week 3, n = 4 sham and n = 6 SNC at week 6.

Complementing the MRI assessments, toe spread and electrophysiological studies were performed to assess functional recovery. Assessment of motor reflex by toe spread scoring indicated that the scores were normal (wide toe spread, score 2) at all time points throughout the experiment for young and old mice submitted to sham operation (Fig. 2a). In the first week after SNC, toe spread reflex was dramatically reduced to score 0 in both young and old animals, indicating deficits in hindlimb sensory-motor function^31,32^. However, whereas toe spread was fully recovered at approximately week 4 after SNC in young mice, it was not fully recovered to score 2 in all old mice by week 6 after injury (Fig. 2a). This indicates that motor reflex recovered more slowly in old animals.

To evaluate the number of motor units in the gastrocnemius muscle after SNC and further validate the model, electromyography recordings were performed weekly for up to 7 weeks. Compound muscle action potential (CMAP) and motor unit number estimation (MUNE) were determined in both operated (SNC) and sham control animals. Significantly reduced MUNE in old compared to young mice was observed at baseline (Fig. 2b); in contrast, no difference in CMAP at baseline was observed (Fig. 2b), underlining the presence of collateral sprouting by surviving healthier motor units that re-innervate vacated neuromuscular junctions^33^. CMAP and MUNE were not impacted by sham operation in both age groups. In contrast, a steep drop in CMAP was observed in young and old mice one week after SNC as a consequence of the loss of muscle innervation subsequent to nerve injury (Fig. 2c). Full CMAP recovery was seen in young mice already at week 5 after nerve injury. In contrast, CMAP recovered only at week 7 in old mice (Fig. 2c).

Sham operation had no impact on MUNE in both age groups, while SNC resulted in a significant drop of MUNE that recovered at week 5 in young mice. Despite that MUNE was already reduced in old compared to young mice at baseline, SNC caused an additional drop that did not recover up to week 7 following the surgery in old animals (Fig. 2d). Sham operation had no impact on MUNE in both age groups. In comparing MRI and electrophysiological parameters, we found that MTR significantly correlated with CMAP and MUNE in both young and old mice (Table 1).

**Table 1:** Comparisons between global MTR and other parameters assessed in young mice in the SNC experiment. n.s. stands for not significant.

Parameter compared to global MTR	Pearson’s coefficient of correlation		Significance p	
	Young mice	Old mice	Young mice	Old mice
CMAP	0.73	0.43	9.3 × 10^−8^	0.008
MUNE	0.73	0.54	9.1 × 10^−8^	3.4 × 10^−4^
Muscle volume	0.38	0.32	0.014	0.055
Muscle CSA	0.41	0.3	0.009	n.s.
Muscle T_2_	−0.66	−0.45	3.6 × 10^−6^	0.006

### Histological analysis of nerve sections showed axon damage and myelin disruption that was recovered over time in young but not in old mice

**Figure 3:**
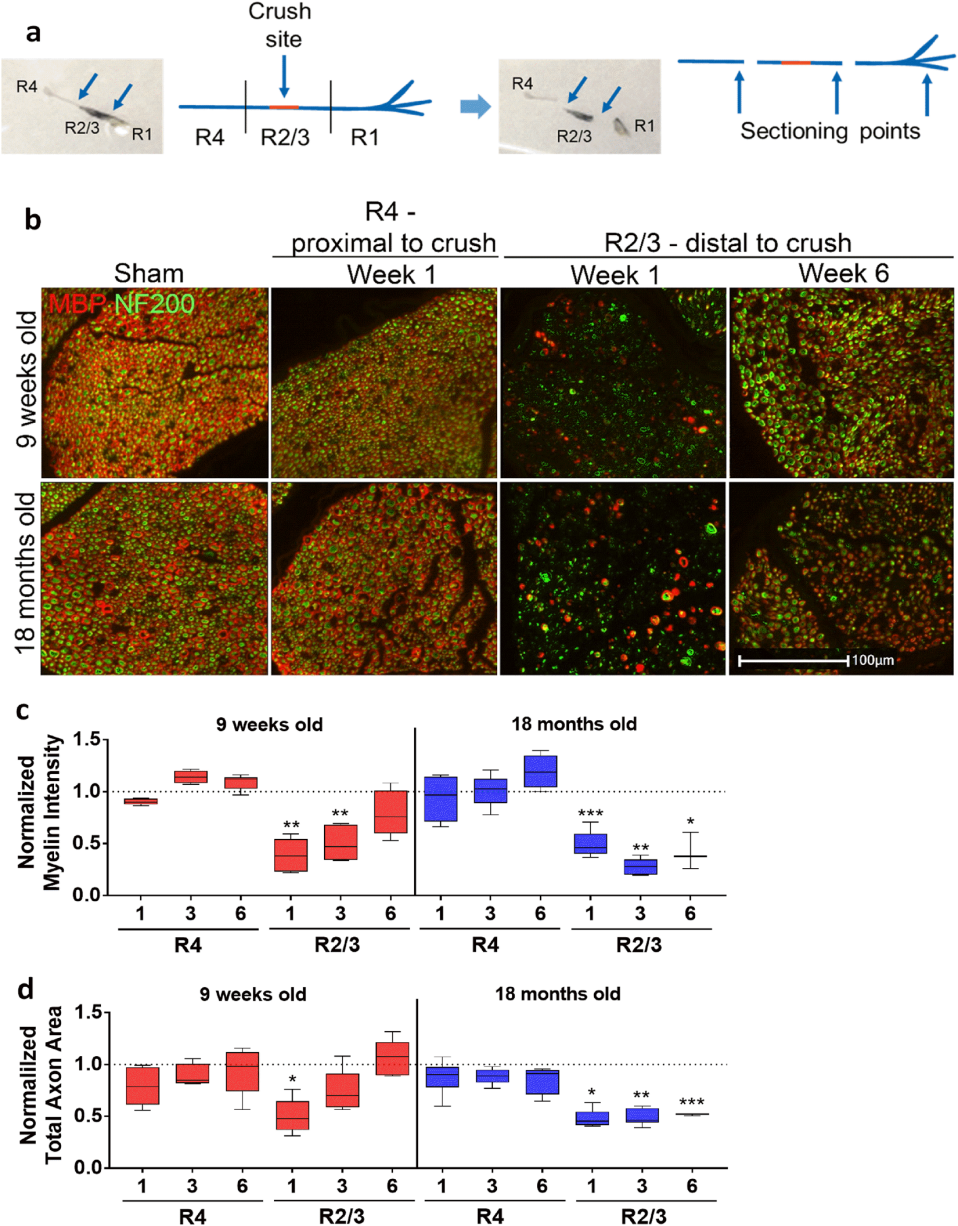
Histology of nerve sections revealed axon damage and myelin disruption that was recovered over time in young but not in old mice. **(a)** Schematic illustration of sciatic nerve subdivisions R4, R2/3 and R1 for histological analysis. Crush site (red) and points of sectioning are indicated. **(b)** Representative images of young and old sciatic nerve transections stained with anti-NF200 (axons, in green) and anti-MBP (myelin, in red) antibodies in regions proximal (R4, at week 1 after SNC) and distal to the crush site (R2/3, at weeks 1 and 6 after SNC). Proximal region R4 of sham-operated nerves have been included for comparison. **(c)** Quantification of myelin intensity in regions R4 and R2/3, normalized over sham-controls (Means ± SEM; *0.01 < p < 0.05, **0.001 < p < 0.01, ***p < 0.001, comparisons between sham and SNC for each region and time point). Abscissa indicates time after SNC (in weeks). **(d)** Quantification of total area occupied by axons in regions R4 and R2/3, normalized over relative sham-controls (Means ± SEM; *0.01 < p < 0.05, **0.001 < p < 0.01, ***p < 0.001, comparison between sham and SNC for each region and time point). Abscissa indicates time after SNC (in weeks). Four sham and 6 SNC mice were analyzed per time point.

To further characterize the recovery following SNC we performed histological analysis of sciatic nerves. The sciatic nerve tissue was subdivided into 3 parts (Fig. 3a), corresponding to regions R1, R2/3 and R4 described above, and stained for markers of the 200 kDa neurofilament protein (NF200) to label the axons as well as of myelin basic protein (MBP) to detect the myelin sheath wrapped around them. Representative images of sciatic nerves are displayed in Fig. 3b to show nerve disruption and recovery after SNC. Images with single channels (either NF200 or MBP) are shown in Suppl. Fig. 2a,b. Regions R1 were excluded from the analysis due to high variability among samples as a consequence of technical difficulties in tissue preparation related to the fact that the distal portion of the mouse sciatic nerve is organized in very small-diameter branches extending in several orientations rendering the proper embedding and transversal sectioning difficult. For all regions and time points analyzed, histological MBP analysis of the nerves showed myelin sheaths organized in the well-known ring-structure around axons in both young and old sham-operated mice. Moreover, NF200-labeled axons were evenly distributed and packed in the nerve bundles. Of note, there was a non-significant trend towards higher myelin content in young compared to old sciatic nerves (Suppl. Fig. 2b,c). However, axon calibers in old sciatic nerves were larger than in young ones, suggesting axon swelling as a result of cytoskeleton disruption, as described in other age-related neurodegenerative diseases^34^ (Suppl. Fig. 2a,c).

In the region R4 of crushed nerves, the portion of the nerve that was located proximal to the crush point, myelin and axons were intact, similarly to what was observed in sham-operated nerves, for both young and old mice. Distally to SNC, in region R2/3, myelin and axons appeared extremely damaged and disorganized at week 1 after injury: the ring structure of myelin disappeared and intense myelin accumulation, resembling debris, was observed in both young and old nerves. Quantification of myelin in regions R4 and R2/3 suggested a full recovery by week 6 in young mice but not in old mice (Fig. 3c). In addition to myelin, axons were damaged by the SNC and lost their organized distribution within the bundle: similarly to myelin, full recovery of axon size was achieved earlier in young than in old mice (Fig. 3d).

### Muscle atrophy was more pronounced on aged muscles and did not fully recover at 6 weeks post SNC

**Figure 4:**
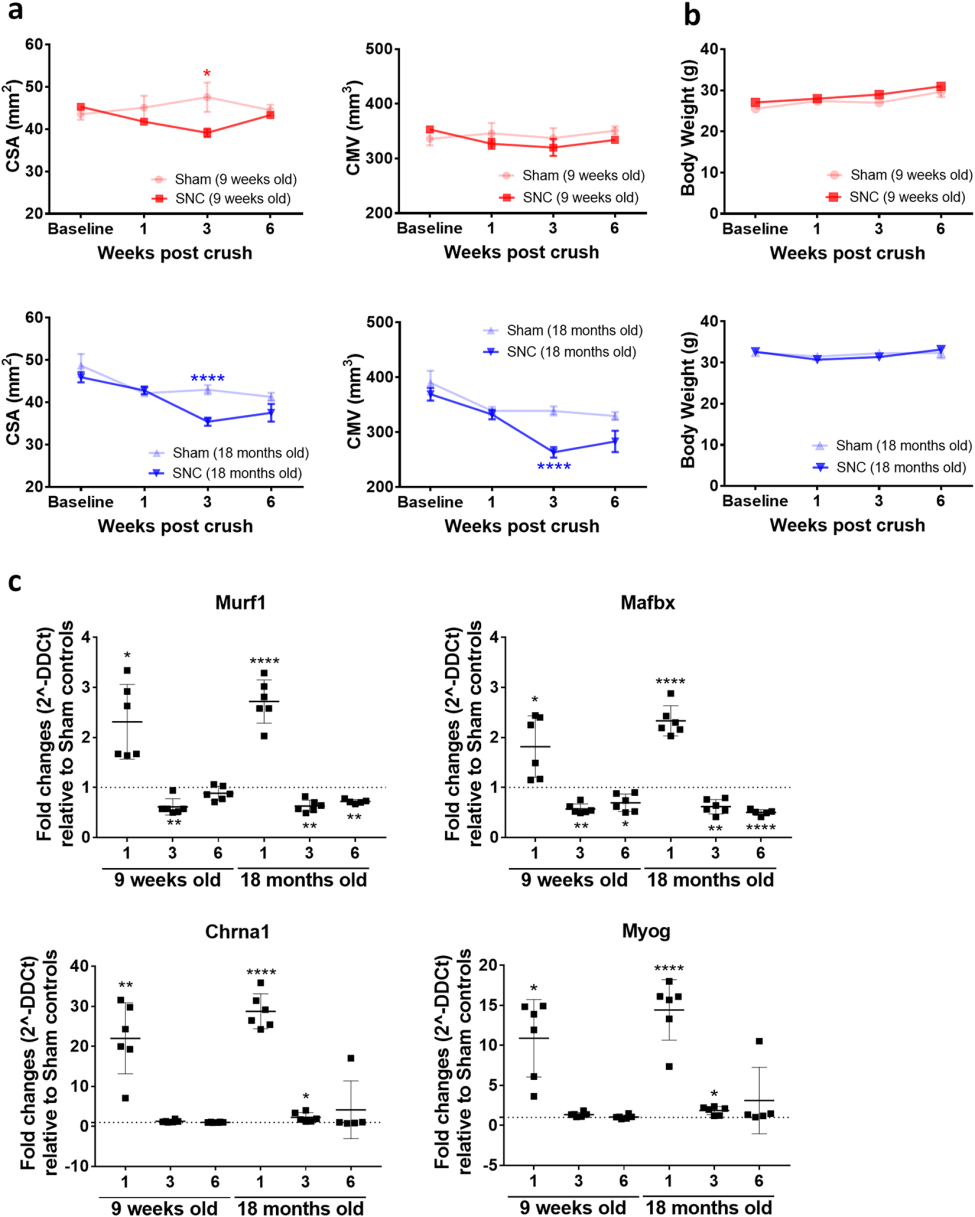
Muscle atrophy was more pronounced on aged muscles and did not fully recover at 6 weeks post-SNC. **(a)** Cross sectional area (CSA, in mm^2^) and calf muscle volume (CMV, in mm^3^) measured by MRI in young and old mice (Means ± SEM; *0.01 < p < 0.05, ****p < 0.0001, comparisons between sham and SNC for each age group). **(b)** Body weight (g) of young and old mice. **(c)** Gene expression analysis of common denervation (Chrna1, Myog) and atrophy (Mafbx, Murf1) markers in gastrocnemius muscle. Dotted line corresponds to relative sham controls (Means ± SEM relative to sham-controls; *0.01 < p < 0.05, **0.001 < p < 0.01, ****p < 0.0001). Number of mice in each age group for MRI and body weight: n = 12 sham and n = 18 SNC at baseline and week 1, n = 8 sham and n = 12 SNC at week 3, n = 4 sham and n = 6 SNC at week 6. Gene analyses were performed on muscles from n = 4 sham and n = 6 SNC mice per time point.

Skeletal muscle atrophy is an important clinical sequela of peripheral nerve injury due to impaired neuro-muscular function. To further complement the nerve investigations, we determined how SNC affected the skeletal muscle of young and old mice by assessing calf muscle volume (CMV) and cross sectional area (CSA) with MRI. After SNC, CSA in the young group gradually declined by approximately 10% up to week 3 but recovered by week 6 (Fig. 4a). CMV did not show any significant change in young muscles. In marked contrast, old mice exhibited a reduction of about 25–30% in both CMV and CSA at week 3 after SNC, thus indicating that aged muscle atrophy was much more severe following disrupted nerve input in comparison to young (Fig. 4a). Interestingly, changes in muscle parameters were found significantly different at week 3, chronologically following changes in the nerve (i.e. MTR) occurring already at week 1 (Fig. 1). No changes in CSA and CMV were observed for both young and old mice in sham-operated hind limbs. Of note, body weight was not affected by SNC (Fig. 4b).

To confirm efficient nerve damage and consequent muscle denervation, we assessed longitudinally gene expression changes of atrophy and denervation markers in the skeletal muscle. Common markers of atrophy (*Murf1*, *Mafbx*) and denervation (*Chrna1*, *Myog*) were greatly upregulated in the gastrocnemius muscle at week 1 after SNC and similarly restored to baseline values in both young and old mice at weeks 3 and 6 after SNC (Fig. 4c). Not surprisingly, changes in gene expression anticipated those observed by MRI, thus suggesting that an activation of the muscle transcriptional machinery as a consequence of nerve damage occurred as early as one week after SNC. This is consistent with the observation that effects on the muscle were detected by MRI only at a later time point.

### Increased T_2_ of muscle in the early phase after SNC consistent with changes in fiber type

**Figure 5:**
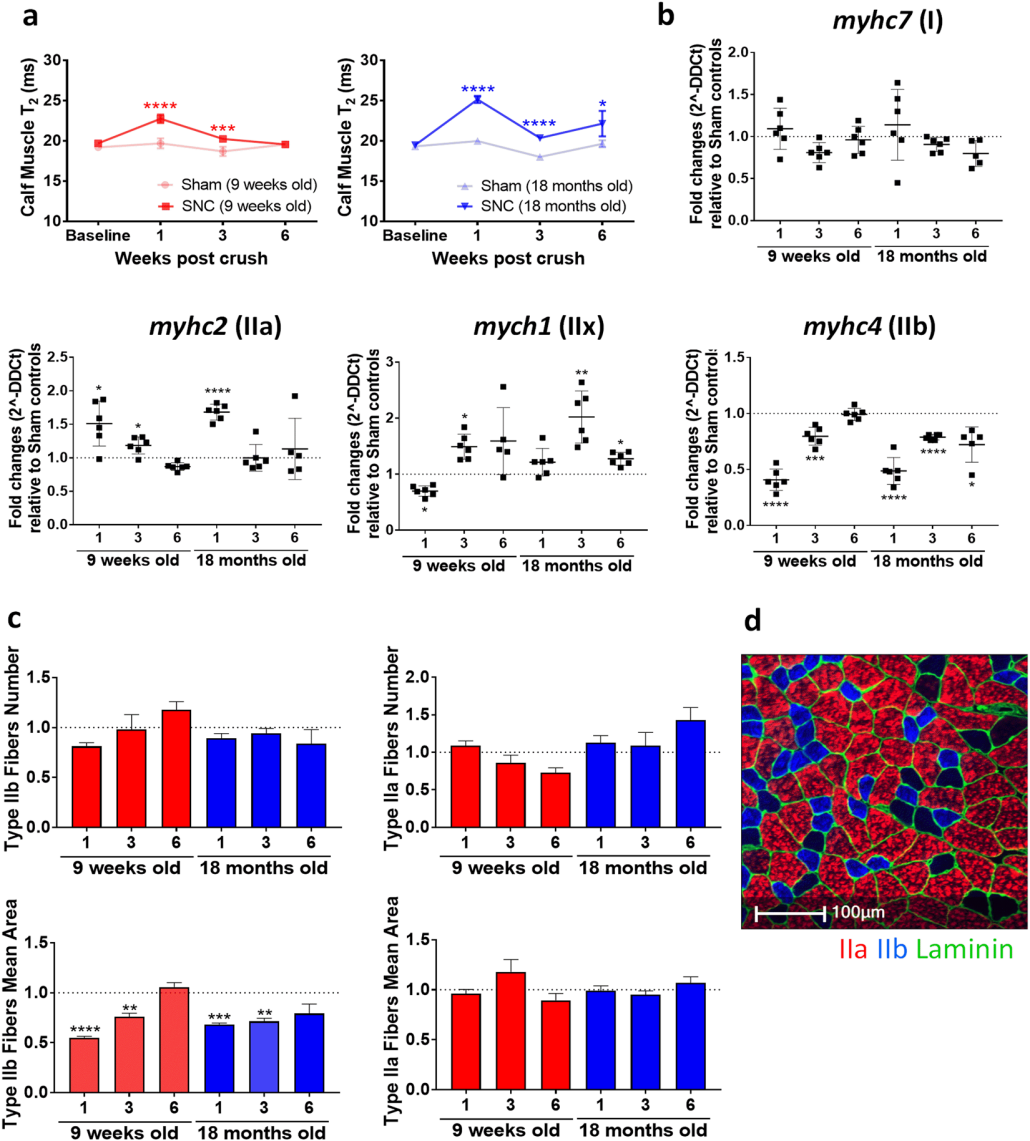
Increased muscle T_2_ in the early phase after SNC consistent with changed fiber type. **(a)** T_2_ (ms) in calf muscles of young and old mice (Means ± SEM; *0.01 < p < 0.05, ***0.0001 < p < 0.001, ****p < 0.0001, comparisons between sham and SNC). **(b)** Gene expression analysis of myosin heavy chain isoforms (*myhc2*, *myhc4*, *myhc7*, *myhc1*) in gastrocnemius muscle of young and old mice. Dotted line corresponds to relative sham controls (Means ± SEM relative to sham-controls; *0.01 < p < 0.05, **0.001 < p < 0.01, ***0.0001 < p < 0.001, ****p < 0.0001). Abscissa indicates time after SNC (in weeks). **(c)** Number and mean area of type IIb and type IIa fibers in transverse sections of gastrocnemius muscle of young and old mice, normalized over the analyzed area and relative to sham-controls (Means ± SEM). Dotted line corresponds to relative sham controls (Means ± SEM relative to sham controls; **0.001 < p < 0.01, ***0.0001 < p < 0.001, ****p < 0.0001. Abscissa indicates time after SNC (in weeks). **(d)** Representative image of a transverse section of gastrocnemius muscle: type IIb and type IIa muscle fibers are labeled and laminin is used to label fibers outline. Number of mice in each age group examined by MRI: n = 12 sham and n = 18 SNC at baseline and week 1, n = 8 sham and n = 12 SNC at week 3, n = 4 sham and n = 6 SNC at week 6. Four sham and 6 SNC mice were examined post-*mortem* per time point.

Further analysis of calf muscle by MRI showed changes in the T_2_ relaxation time after SNC, in a region distant from the site of the surgery. Increased T_2_ of calf muscle was detected at weeks 1 and 3 after SNC in both young and old mice compared to sham controls. However, at week 6, muscle T_2_ in young animals had returned to normal values, while T_2_ in old mice was still significantly increased (Fig. 5a).

To address a possible fiber type switch upon SNC, quantitative polymerase chain reaction (qPCR) and histological analyses were performed on the gastrocnemius muscle. The gene expression marker for slow oxidative type I fibers, *myhc7*, was unchanged in both young and old mice for all time points after SNC. However, *myhc1*, a marker of type IIx fibers, was increased at week 3 after SNC in muscles of both young and old animals, but remained significantly increased at week 6 in muscles of old mice only, underlining an impaired or slower recovery in the old group. Moreover, *myhc2*, a marker for type IIa fibers, was significantly increased already at week 1 after SNC and gradually declined to normal levels by week 6 in both young and old mice (Fig. 5b). In contrast, the gene expression marker for type IIb fibers, *myhc4*, was significantly decreased at week 1 after SNC and gradually recovered up to week 6 in young mice. A decrease in *myhc4* in the early phase after SNC followed by an incomplete recovery was observed in the old mice (Fig. 5b). Histological analysis of type IIa and IIb fibers in transversal sections of gastrocnemius muscle confirmed the qPCR data: although there was only a non-significant trend towards reduction in the number of type IIb fibers following SNC, the total area of IIb fibers was significantly lower at weeks 1 and 3 after the crush in young and old animals (Fig. 5c). At week 6, the area of type IIb fibers in the muscle of crushed limbs in old mice was still below that of sham limbs, although the effect was not significant. In contrast, changes in type IIa fibers number and mean area did not reach statistical significance at all the analyzed time points. A representative image of type IIa and IIb fibers staining in gastrocnemius muscle is shown in Fig. 5d. Altogether, the *in vivo* T_2_ relaxation time and the post-*mortem* qPCR and histology results suggest type IIb muscle fibers atrophied following SNC then gradually recovered over time.

MTR was weakly correlated to muscle volume and CSA in young mice (Table 1). In old animals, MTR was only weakly correlated to muscle volume. However, MTR and muscle T_2_ were negatively correlated in young and old mice.

### A second model of local demyelination: Injection of lysolecithin (LCP) in rats

**Figure 6:**
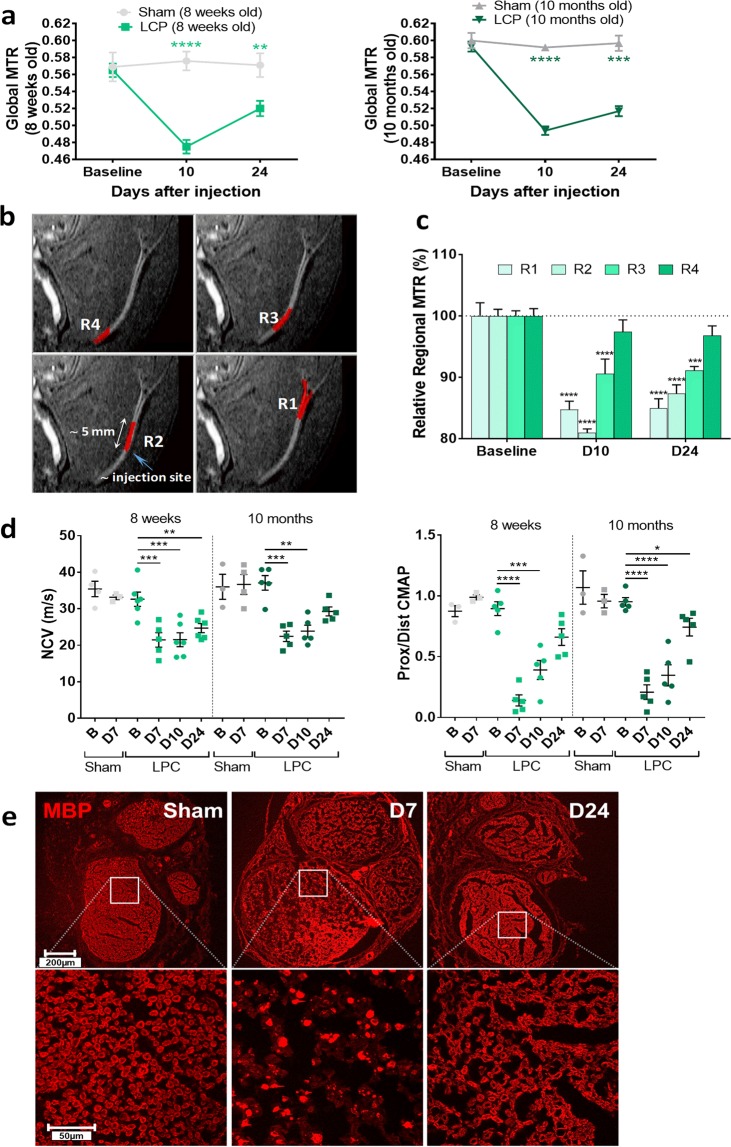
Sciatic nerve demyelination induced by lysolecithin (LCP) in rats. **(a)** Global MTR at baseline and following LCP injection in young and adult rats (Means ± SEM; **0.001 < p < 0.01, ****p < 0.0001, comparisons between sham and LCP groups at each age). **(b)** Representative MRI images with sub-regions R1-4 of sciatic nerve highlighted in red. **(c)** Regional MTR analyzed at baseline and at days 10 and 24 (D10 and D24) after LCP injection in young rats and expressed as relative to baseline values for each region (Means ± SEM; *0.01 < p < 0.05, ***0.0001 < p < 0.001, ****p < 0.0001, comparisons to baseline values for each region). **(d)** Nerve Conduction Velocity (NCV) and the ratio between proximal and distal CMAP (prox/dist CMAP) measured by EMG in young (8 weeks) and adult (10 months) rats at baseline (B) and at day 7, 10 and 24 (D7, D10, D24) after LCP injection in the sciatic nerve. Sham-control rats were also analyzed at baseline (B) and at day 7 (D7). Means ± SEM; *0.01 < p < 0.05, **0.001 < p < 0.01, ***0.0001 < p < 0.001, ****p < 0.0001. **(E)** Representative images of Myelin Basic Protein (MBP) in sciatic nerve transections of sham-operated and LCP-treated young rats at day 7 and 24 after LCP injection (D7, D24). Top panels: whole nerve section; bottom panels: detailed images of nerve portions. Number of rats: n = 4 sham and n = 6 LCP for each age group.

To further observe changes in myelin density in a second model of peripheral nerve damage, we induced demyelination via lysolecithin (LCP) injection. Following local injection of LCP in the sciatic nerve to induce demyelination, the recovery phase was monitored longitudinally by MRI and electrophysiological readouts. LCP-induced demyelination is thought to involve the action of recruited macrophages or microglia in the central nervous system, which phagocytose nearby myelin. Indeed, a decrease by 15.9% and 16.7% in global MTR was detected 10 days after LCP administration in young (8 weeks old) and adult rats (10 months old), respectively (Fig. 6a). At day 24 post-LCP, MTR in both age groups was still significantly reduced with respect to baseline values, by 7.9% and 12.7% in young and adult animals, respectively (Fig. 6a). Representative MRI images of rat sciatic nerves subdivided in regions R1-R4 are shown in Fig. 6b. Regional analysis in adult nerves demonstrated a similar reduction in MTR in the nerve regions (R1, R2) adjacent to the injection site of LCP. Closer to the body center, there was a less pronounced but still significant MTR reduction in region R3 compared to R1,R2, while only a trend in MTR reduction was observed in region R4. At day 24, MTR was still significantly reduced in regions R1-R3 (Fig. 6c and Suppl. Fig. 3).

Electrophysiological assessments were also performed at days 7, 10 and 24 following LCP injection to monitor the recovery phase. In young rats (8 weeks old), nerve conduction velocity (NCV) and proximal/distal CMAP (prox/dist CMAP) were significantly reduced at days 7 and 10 after LCP application (Fig. 6d). At day 24, NCV was not fully recovered yet while prox/dist CMAP was no longer significantly different compared to baseline (Fig. 6d). In adult rats (10 months), prox/dist CMAP was significantly reduced at days 7, 10 and 24 after LCP injection; NCV was significantly changed only at day 7 and 10 and recovered at day 24 (Fig. 6d).

Similarly to the SNC model, histological analysis of sciatic nerve sections revealed the presence of highly disorganized myelin, mostly resembling myelin debris, at day 7 after LCP injection (Fig. 6e). In addition, swelling of the nerve bundles has been observed at day 7 post-LCP, which disappeared at day 24 (Fig. 6e). This effect could be due to the injection volume itself perturbing the nerve or to edema caused by demyelination. Visual inspection of histological sections indicated that, at day 24, albeit not restored to normal levels, myelin content was higher than at day 7 (Fig. 6e).

### Calf muscle changes induced by LCP injection

**Figure 7:**
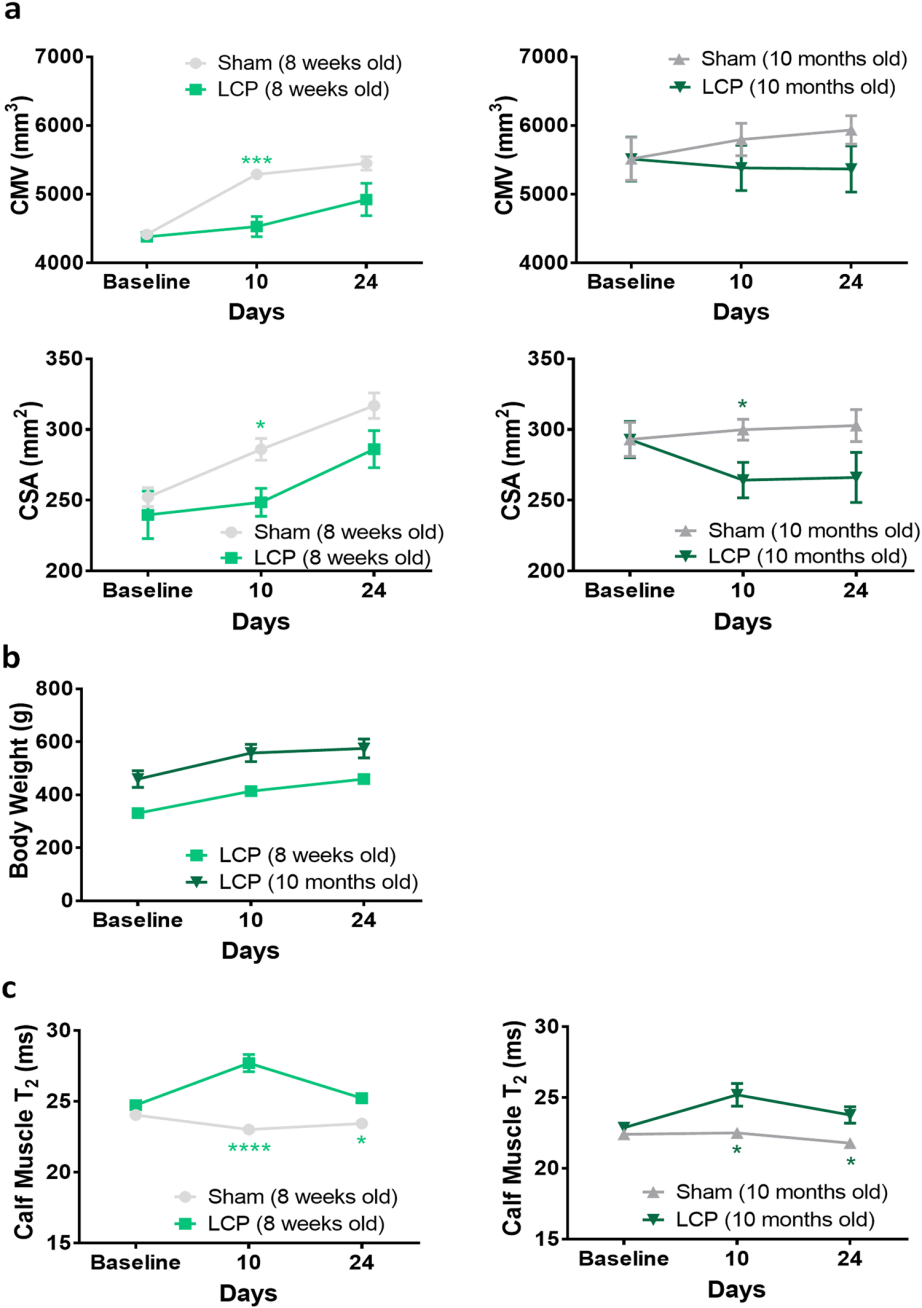
Effects of LCP on calf muscle. **(a)** Calf muscle volume (CMV, in mm^3^) and cross sectional area (CSA, in mm^2^) measured by MRI in calf muscles of sham-control and LCP-treated young and adult rats, at baseline and at days 7, 10 and 24 following LCP injection (means ± SEM, *p < 0.05, ***p < 0.001, comparisons between sham and LCP groups). **(b)** Body weight (g) of young and adult rats, monitored at baseline and at day 10 and 24 following LCP injection. Means ± SEM. **(C)** T_2_ in calf muscles of young and adult rats, at baseline and at days 7, 10 and 24 after LCP injection (Means ± SEM; *p < 0.05, ****p < 0.0001, comparisons between sham and LCP groups). Number of rats: n = 4 sham and n = 6 LCP for each age group.

Calf muscle volume (CMV) and CSA of the hind limbs of young sham-operated rats increased significantly during the experiment period, reflecting the physiological growth of the animals, whereas for adult sham rats there was only a trend towards an increase in these parameters (Fig. 7a). Despite the fact that CMV and CSA increased significantly also in the LCP-treated hind limbs of young rats, the rate of increase was significantly lower when compared to the contralateral side (Fig. 7a). For adult rats, both CMV and CSA were reduced in LCP-treated compared to contralateral hind limbs (Fig. 7a). No reduction in body weight was observed after LCP injection (Fig. 7b).

T_2_ relaxation time was also measured in calf muscle of both young and adult rats, in a region distant from the site of LCP administration. Injection of LCP resulted in a significant increase of muscle T_2_ by 10–12% on day 10 for both ages (Fig. 7c); however, the relaxation time was almost completely recovered by day 24.

Altogether, these data suggest that the LCP model displayed a phenotype similar to the one observed in the SNC model, in both nerves and muscles. Moreover, only partial differences were observed in young compared to adult rats, indicating that older animals should be included for the detection of more pronounced age-related effects.

## Discussion

Peripheral nerve injury has been classified on the basis of damage to Schwann cells/myelin, axons, endoneurium, perineurium, and epineurium (grades 1–5, respectively)^35^. Electromyography and nerve conduction studies are considered as the gold standard in the assessment of peripheral nerve lesions^3^. However, the evaluation of the severity of a nerve lesion and assessment of early nerve regeneration by electrophysiology remain difficult, as such assessments have limited ability to distinguish different grades of injuries in the acute and subacute stages^36^. Determining the site and degree of the nerve lesion is important for patient management especially in the early phase after injury. Since skeletal muscles atrophy after denervation and axons regenerate slowly, nerve injuries need to be accurately graded in order to expedite surgical or pharmacological intervention to prevent permanent loss of function. In addition, there is a need for noninvasive diagnostic testing to monitor the effects of various novel approaches on nerve repair ^37–39^.

Diffusion tensor imaging (DTI) has shown great promise to assess nerve repair^21^ but remains a technically challenging technique especially in the preclinical setting with small rodents. Measurement times of 12 hours of excised rat nerves^24^ demonstrate the challenge of incorporating DTI into routine testing in small rodent models. Alternatively, Gd-DTPA^40,41^ or gadofluorine M enhancement^18,19,42^ have been explored as a means to analyze peripheral nerve injury models in rats. Due to potential safety issues with Gd-based contrast agents^43,44^, avoiding their use when testing new therapies might be advisable.

In view of the desire to be able to capture longitudinal measurements in the same animal, MTR was assessed in the present work as a potential non-invasive MRI marker of peripheral nerve injury in mouse models. An advantage of the approach is that it does not require the administration of contrast material. Similar to diffusion MRI, MTR is sensitive to changes in myelin density that occurs due to demyelination or axonal loss^6,7^, and a short examination time is feasible. At baseline, MTR was lower in old compared to young mice, a result that is consistent with an age-dependent decrease of sciatic nerve MTR detected in human volunteers^45^. A decrease in MTR is most likely the result of an age-related damage in myelin lipids, resulting in a decrease in myelin concentration as suggested by animal studies^46^ and imaging investigations in healthy individuals showing a decrease in fractional anisotropy with age revealed by DTI, indicating a decrease in myelination with age^47^. Nevertheless, decreased MTR signal has been also associated with changes in axonal integrity^48–51^. In our study, histological analyses of sham-operated animals revealed only a trend towards lower myelin levels in the nerves of old compared to young mice (Suppl. Fig. 2c). However, axons caliber in old sciatic nerves was bigger than in young ones (Suppl. Fig. 2c). Axon swelling as a result of cytoskeleton disruption has been described in age-related neurodegenerative diseases or injury^34^. Moreover, areas of decreased MTR in MS lesions were shown to contain swollen axons^49^. Experimental animal models as well as molecular and morphological analysis of chronic MS brains have demonstrated in the course of the pathology a malfunction of ion channels and pumps that maintain axoplasmic sodium/potassium (Na/K) gradients during nerve conduction^52,53^. An imbalance of Na/K exchange, increased axoplasmic calcium and reduced adenosine triphosphate production were all shown to contribute to a cascade of events leading to swelling and ultimately death of demyelinated axons. Therefore, the reduced MTR assessed here in old compared to young nerves before SNC might have primarily reflected axon swelling.

In 1972, Hall was the first to demonstrate the use of LCP to produce rapid demyelination in the white matter of the adult mouse spinal cord^54^. Since then, LCP has been extensively used to study de- and remyelination in the nervous system^55,56^. MTR changes in both SNC and LCP-induced sciatic nerve injury correlated with electrophysiological parameters (MUNE, NCV) as well as with myelin content assessed by histology. Consistent with the need for axon regrowth before myelination, the repair phase following SNC in young mice was characterized by a recovery of MTR in areas proximal to the crush while the MTR in distal regions (encompassing the sciatic nerve bifurcation) did not recover to baseline values at week 6 post-crush. In old mice, MTR at week 6 did not recover in a larger area, comprising the distal and also more proximal areas of the sciatic nerve. This pattern was confirmed by histological analysis of myelin and is consistent with delayed nerve regeneration in the aged^57^. For LCP injection, both global and regional MTR values were still significantly reduced at 24 days after toxin administration. Taken together, these data show that both models provide a good window for testing compounds aiming at accelerating remyelination, using MTR as metrics for myelin content. The MTR changes were not too different in the axonotmetic grade of injury (SNC) and the neuropraxia grade injury (LCP model). Perhaps this is because the crush approximates axonal injury that is on the ‘weaker’ side of the axonotmetic injuries (2 on the injury scale) and therefore the window to differentiate from the LCP (1 on the injury scale) may be too small. Of note, using comparable acquisition parameters, the degree of MTR reduction (∼15%) in the early phase following nerve injury by SNC or LCP injection was of the same order of magnitude as that obtained for demyelination induced by cuprizone in the central nervous system^29^.

Dortch *et al*.^25^ demonstrated that MTR measurements of the sciatic nerve correlated with clinical disability in CMT disease patients. The imaging protocols and data analysis pipelines resulted in highly reproducible MTR measurements. These observations suggested MTR of proximal nerves to be a valuable biomarker, especially as distal nerves are often fully degenerated in CMT disease and proximal nerves are difficult to access via conventional techniques. Moreover, Mekle *et al*.^58^ demonstrated that MTR provides a means for high spatial resolution imaging and tracking of peripheral foot nerves. Its methodology is directly applicable on standard clinical MRI scanners. MTR of foot nerves could serve as a biomarker for early detection, monitoring, and prognosis of systemic pathologies, such as diabetes, traumatic injuries, and metabolic diseases. Our results further strengthen the translational character of MTR, pointing to its value as readout in preclinical and clinical studies of therapies aiming to repair injured nerves.

Adopting MTR as a parameter to monitor nerve regeneration has limitations. Does *et al*.^59^ showed in isolated amphibian sciatic nerves that relaxation times (T_1_, T_2_) may impact the assessment of MTR. Thus, MTR is not an absolute measure of myelin content, being notably affected by T_1_ and inhomogeneous profiles of radiofrequency excitation. Alternatives were developed to minimize this impact, for instance the so-called “MTsat” method that corrects for T_1_ variation using T_1_- and proton density-weighted scans^60^ or quantitative magnetization transfer (qMT) providing more accuracy by modeling magnetization transfer (MT) exchange pools^61^ and/or using special preparation methods^62^. Drawback of these approaches are the long acquisition times, making it challenging to adopt them in routine pharmacological studies involving a large number of mice. By their very nature, *in vivo* pharmacological investigations always involve the comparison of vehicle and compound-treated animals. Therefore, even if a systematic error is made when assessing MTR, all measurements will be affected. In this work we opted for MTR as a rapid readout, and preferred to use additional measurement time to examine the muscle as well. On the other hand, the MT signal may also be affected by factors like macrophage infiltration, activated microglia, inflammation/edema^27,63^. Possible effects of inflammation on the MTR assessments might be more prominent in the early phase after the injury. Moreover, myelin debris could have also contributed to the MTR determination^64^. A freeze/fracture study on macrophages from LCP-induced lesions in the spinal cord of rats showed that phagocytosed myelin initially retained its lamellar structure within vacuoles before eventual lysosomal digestion forms filled vacuoles^65^. This retained lamellar structure within macrophages might impact MTR assessments dependent on exchange mechanisms that further depend on the restricted water environment created by the structure of intact myelin. Careful histological examination is therefore important in order to well validate the readouts before embarking on routine assessments of compound effects in the models. It is noteworthy that in the present work, we performed multislice MTR imaging. Because of possible inter-slice MT effects^66^, some bias might have been introduced into our assessments.

Analysis of calf muscle by MRI showed changes in the T_2_ relaxation time after SNC, in a region distant from the site of the surgery. Earlier studies revealed that T_2_ of a slow-twitch muscle is longer than that of a fast-twitch muscle^67,68^. Furthermore, it has been shown that the T_2_ of fast-twitch muscle increases with age due mainly to increased extracellular space, reflecting age-related type II fiber atrophy^69^. Type II fast-twitch glycolytic muscle fibers are generally more vulnerable than slow-twitch oxidative (type I) fibers. At the molecular level, fiber-specific atrophy appears to be attributed to different signaling pathways, and most of them are relevant to abnormal protein degradation, including proteasomal and lysosomal pathways^70^. One week after nerve injury by SNC, we observed significantly reduced myosin heavy chain (MYHC)-IIb mRNA levels and type IIb fibers mean areas by histological analysis in gastrocnemius muscle, that gradually recovered over time. Therefore, together with the MRI data, our gene expression and protein analyses of fiber types indicated a reduction in type IIb fast-twitch glycolytic fibers as a consequence of nerve damage. Interestingly, type IIb fibers returned to normal levels approximately at week 6 after SNC, thus suggesting that innervation was restored, nerve fibers gradually re-established a contact with muscle endplates and restored the normal MYHCs expression. Nevertheless, fiber type-specific vulnerability is not yet fully understood and requires further studies.

In summary, the present results demonstrate MTR as a non-invasive MRI marker of peripheral nerve injury in mouse models. The measurements are reproducible and fast. The parameter is of translational value, since on one hand MTR measurements of the sciatic nerve correlated well with histological assessment of myelin as shown here while on the other hand MTR correlated with clinical disability in Charcot-Marie-Tooth patients as demonstrated by Dortch *et al*.^25^. Assessing additional parameters in the muscle enables a more comprehensive analysis of the consequences of nerve injury. Overall, MRI of peripheral nerves has potential to support the testing and development of therapies aiming to restore nerve function after injury.

## Materials and Methods

Animal handling, care, and experimental use were in line with the Swiss federal laws for animal protection. The studies described in this report were approved by the Cantonal Veterinary Authorities of Basel, Switzerland (licenses BS-2804 and BS-2874).

### Animals

Male C57BL/6JRj mice (9 week-old n = 30, 18 month-old n = 30 at the beginning of the study) and Wistar rats (8 week-old n = 10, 10 month-old n = 10 at the beginning of the study) were purchased from Janvier Laboratories (Le Genest-Saint-Isle, France) and from Charles River Laboratories (Sulzfeld, Germany) respectively. Animals were allowed to adapt for 7 days prior to the start of an experiment. They were housed under standard conditions (temperature 20–24 °C, relative humidity minimum 40%, light/dark cycle 12 h) in IVC racks (max. 4 animals/cage) and given access to standard food (Kliba Nr. 3893.025; Kliba-Provimi, Kaiseraugst, Switzerland) and water ad libitum.

### Mouse sciatic nerve crush (SNC)

Animals were induced into anesthesia at a dose of 4% isoflurane (Abbott, Cham, Switzerland) and then maintained by continuous inhalation of 1.5–2% isoflurane on a heated surface (approximately 15 min). The skin was incised posterior and parallel to the left femur. The left sciatic nerve was exposed at mid-thigh and then crushed for 15 seconds proximal to its branching using a Dumont 5 forceps^71^. Powdered charcoal was used to mark the site of crush. Following the crush, the nerve was repositioned and the muscle closed with a dissolvable suture (0.7 gauge) and the skin incision closed with wound closure clips. Carprofen (Rimadyl, Pfizer, Zurich, Switzerland, 5 mg/kg) was administered subcutaneously for post-operative pain control. Mice were then allowed to recover from the anaesthesia and placed in their cages. In sham-operated animals the skin was incised, the muscles were pulled apart and the nerve was lifted gently without performing any crush.

### Rat nerve demyelination by lysolecithin (LCP)

Surgery was performed under isoflurane anaesthesia (approximately 20 min) on a heated surface, as described above for SNC. The skin was incised posterior and parallel to the left femur. The left sciatic nerve was exposed at mid-thigh and 5 μl of 20 mg/ml LCP (L4129, Sigma-Aldrich, Buchs, Switzerland) solution (in physiological saline; 0.9% NaCl) was injected proximal to the branching point using a Hamilton syringe (Sigma-Aldrich) with 30 gauge needle. The needle was inserted parallel to the nerve with the bevel upwards and the solution was slowly injected over the course of about 1 minute. The nerve was repositioned and the muscle then closed with a dissolvable suture (0.6–0.7 gauge) and the skin incision closed with wound closure clips. Carprofen (5 mg/kg) was administered subcutaneously for post-operative pain control. Rats were then allowed to recover from the anaesthesia and placed in their cages.

### Magnetic resonance imaging (MRI)

Measurements were performed with a Biospec 70/16 spectrometer (Bruker Medical Systems, Ettlingen, Germany) operating at 7 T. The operational software of the scanner was Paravision 5.1 (Bruker). Images were acquired from isoflurane anesthetized, spontaneously breathing animals using a 40 mm diameter (model A300HBEL001, Rapid Biomedical, Würzburg, Germany) or a 72 mm diameter circularly polarized coil (model V-HQ-070-01153-001 V01, Rapid Biomedical) for radiofrequency excitation and detection in mice or rats, respectively. Neither cardiac nor respiratory triggering was applied. Following a short period of introduction in a box, mice or rats were maintained in anesthesia with respectively 1.5% or 2.5% isoflurane in oxygen, administered via a nose cone. During MRI signal acquisitions, animals were placed in prone position in a cradle made of plexiglas, the body temperature was kept at approximately 37 °C using a heating pad, and the respiration was monitored. The animals were positioned in such a way that both legs could be measured simultaneously.

Following acquisitions were performed:

1. T_2_-weighted, multislice RARE (Rapid Acquisition with Relaxation Enhancement) for determining the anatomical orientation of the nerves. Parameters: effective echo time (TE) 42.8 ms, repetition time (TR) 3800 ms, RARE factor 16, 8 averages. Gauss512 pulses of duration/bandwidth 2 ms/1370 Hz and 1.27 ms/1270 Hz, with a shape consisting of 512 amplitude and phase values truncated at 1%, were used for radiofrequency (RF) excitation and refocusing, respectively. Fat suppression was achieved by a hermite pulse of 5.2 ms/1039 Hz duration/bandwidth followed by a 2-ms-long gradient spoiler. Anatomical parameters (expressed in parentheses for rats) were: field-of-view (FOV) 30 × 24 mm^2^ (60 × 60 mm^2^), matrix size 256 × 128, pixel size 0.117 × 0.188 mm^2^ (0.234 × 0.469 mm^2^), slice thickness 0.4 mm (0.8 mm), 13 adjacent slices. Acquisition time 4 min 3.2 s.
2. Multislice FLASH (Fast Low-Angle Shot) acquisition for MTR determination: TE 2.8 ms, TR 336 ms, 4 averages, anatomical parameters as for the RARE sequence. A hermite pulse of 0.9 ms/6000 Hz duration/bandwidth and flipangle 30° was used for RF excitation. MTR contrast was introduced by a gauss pulse of 15 ms/182.7 Hz duration/bandwidth applied with radiofrequency peak amplitude of 7.5 µT and an irradiation offset of 1500 Hz. The acquisition was then repeated with the same parameters but without the introduction of the MTR contrast. MTR was then computed using the formula
  1 $${\rm{MTR}}=\frac{{{\rm{S}}}_{{\rm{P}}}-{{\rm{S}}}_{{\rm{MTR}}}}{{{\rm{S}}}_{0}}$$
  where S_0_ and S_MTR_ represent respectively the signal intensities in the FLASH acquisitions without and with the introduction of the MTR contrast. The total acquisition time for both data sets was of 4 min 18.4 s
3. Fat and water separated images of the lower hind legs were acquired with an iterative decomposition of water and fat with echo asymmetry and least-squares estimation (IDEAL) technique with a gradient-echo imaging technique^72^ for assessment of calf muscle volume: TR 604.7 ms, TE’s 5.018, 5.507, and 5.997 ms, flipangle 40°, 4 averages, FOV 70 × 60 mm^2^, matrix size 512 × 128 (reconstructed to 128 × 128), slice thickness 1 mm, 48 adjacent slices, total scan time 15 min 29 s.
4. Spin-echo sequence for T_2_ assessment of calf muscle: TR 2500 ms, TE in multiples of 11 ms ranging from 11 to 176 ms (16 values). Hermite pulses of duration/bandwidth 3.4 ms/1600 Hz and 3.1 ms/1052 Hz were used for RF excitation and refocusing, respectively. Fat suppression as described above for the RARE sequence. Anatomical parameters were (for rats in parentheses) FOV 25 × 25 mm^2^ (60 × 60 mm^2^), matrix 256 × 192, pixel size 0.098 × 0.13 mm^2^ (0.234 × 0.312 mm^2^), slice thickness 1 mm (2 mm), 3 slices.

MTR and T2 assessments were performed using the Paravision software. As both sequences had the same anatomical parameters, the choice of the regions-of-interest (ROIs) for MTR evaluations was performed on the RARE images and then transferred to the FLASH images. T2 values were determined by fitting the curves related to the exponential signal decays obtained by assessing the mean signal intensity in the same ROIs in the images corresponding to the 16 different TE’s. Muscle volume was computed on water and fat separated images using in house developed software based on Matlab (The MathWorks GmbH, Bern, Switzerland).

### Motor unit number estimation (MUNE), compound muscle action potential (CMAP) and nerve conduction velocity (NCV) assessments

Electrophysiological assessments were performed on isoflurane-anesthetized animals using a recording unit of ADInstruments (Oxford, UK), following the procedure described by Arnold *et al*.^73^. Briefly, the sciatic nerve was stimulated via monopolar needle electrodes placed intramuscularly in the left gluteal region, at the sciatic notch (P1). A recording (active) monopolar electrode was placed in the ventral region of the left leg gastrocnemius. A non-active, monopolar electrode was placed in the heel of the left hindlimb.

#### CMAP

Initially, the nerve was stimulated by a single pulse of 0.5 ms duration. The current (in mA) was increased to achieve maximum CMAP. The current achieving the maximum CMAP (I-max) was recorded.

#### MUNE (gastrocnemius)

Following CMAP assessment, the pulse duration was decreased to 0.01 ms. The average single motor unit potential (SMUP) size was determined with an incremental stimulation technique^74^. To obtain incremental responses, a submaximal stimulation at a frequency of 1 Hz was delivered while increasing the current in small steps (typically 0.03 mA) to obtain the minimal all-or-none responses. The current was then gradually increased (in steps of approximately 0.03 mA) to obtain evoked responses to a level of about a tenth of the CMAP. Incremental increases in nerve stimulation intensity generated an envelope of evoked responses, each step representing activation of single motor axons that was serially added to the maximum evoked response. The number of evoked responses from each animal was calculated as the median of the estimates of steps from 4 individuals.

The average SMUP was calculated by dividing the incremental response by the number of steps. MUNE was then calculated as follows:

$${\rm{MUNE}}=\frac{{\rm{maximal}}\,{\rm{compound}}\,{\rm{muscle}}\,{\rm{action}}\,{\rm{potential}}\,({\rm{CMAP}})\,{\rm{amplitude}}}{{\rm{average}}\,{\rm{single}}\,{\rm{motor}}\,{\rm{unit}}\,{\rm{potential}}\,({\rm{SMUP}})\,{\rm{amplitude}}}$$

#### NCV

a single pulse of 0.5 ms duration was used to stimulate the sciatic nerve, via monopolar needle electrodes, first placed intramuscularly in the left gluteal region, sciatic notch (proximal site CMAP) and, secondly, in the distal position (gastrocnemius, distal site CMAP). The recording electrode was placed in the plantar (ventral) of the foot. NCV was then calculated as follows:

$${\rm{N}}{\rm{C}}{\rm{V}}({\rm{m}}/{\rm{s}})=\frac{{\rm{d}}{\rm{i}}{\rm{s}}{\rm{t}}{\rm{a}}{\rm{n}}{\rm{c}}{\rm{e}}\,{\rm{b}}{\rm{e}}{\rm{t}}{\rm{w}}{\rm{e}}{\rm{e}}{\rm{n}}\,{\rm{s}}{\rm{t}}{\rm{i}}{\rm{m}}{\rm{u}}{\rm{l}}{\rm{a}}{\rm{t}}{\rm{i}}{\rm{o}}{\rm{n}}\,{\rm{s}}{\rm{i}}{\rm{t}}{\rm{e}}{\rm{s}}\,({\rm{p}}{\rm{r}}{\rm{o}}{\rm{x}}{\rm{i}}{\rm{m}}{\rm{a}}{\rm{l}}-{\rm{d}}{\rm{i}}{\rm{s}}{\rm{t}}{\rm{a}}{\rm{l}},{\rm{m}})}{{\rm{l}}{\rm{a}}{\rm{t}}{\rm{e}}{\rm{n}}{\rm{c}}{\rm{y}}\,{\rm{p}}{\rm{r}}{\rm{o}}{\rm{x}}{\rm{i}}{\rm{m}}{\rm{a}}{\rm{l}}\,{\rm{s}}{\rm{i}}{\rm{t}}{\rm{e}}-{\rm{l}}{\rm{a}}{\rm{t}}{\rm{e}}{\rm{n}}{\rm{c}}{\rm{y}}\,{\rm{d}}{\rm{i}}{\rm{s}}{\rm{t}}{\rm{a}}{\rm{l}}\,{\rm{s}}{\rm{i}}{\rm{t}}{\rm{e}}\,({\rm{s}})}$$

### Toe spread assessment

To assess motor function recovery, the toe spread reflex was scored. A narrowed toe spread has been described following nerve crush that may be indicative of deficits in hind limb sensory motor function^31,32^. Mice were covered with a piece of cloth and briefly lifted by the tail for the observation of the toe spread in the hindpaws. The toe spread reflex was scored according to the description by Ma *et al*.^75^: 0 - no spreading, 1 - intermediate spreading, 2 - full toe spreading. A mean score was determined for each group of animals at different time points.

### Histology

At the end of the study, animals were euthanized with an overdose of isoflurane followed by decapitation. The central gastrocnemius muscle and sciatic nerve were harvested for histological analysis. Immediately after collection, muscles were embedded in OCT (optimal cutting temperature) compound (Tissue-Tek, Sakura Finetek Europe, Alphen aan den Rijn, The Netherlands) in vapors of isopentane, pre-chilled in liquid nitrogen. Cryosections were prepared using a Cryocut 1800 cryostat (Leica Biosystems, Eisfeld, Germany) at 10 µm and placed on Superfrost Plus microscope slides (Thermo Fisher Scientific, Reinach, Switzerland).

Sciatic nerve samples were placed on a small piece of transparent plastic and fixed in 10% neutral buffered formalin (NBF) solution (HT501128, Sigma-Aldrich, Buchs, Switzerland) for 6 hours. Sciatic nerves samples were then processed for paraffin embedding by dehydration through increasing ethanol series and paraffin infiltration (Paraplast Plus®, P3683, Sigma-Aldrich). Before paraffin embedding, sciatic nerves were cut in three parts: proximal R4, middle R2/3 and distal (including the branching point into tibial and peroneal nerves) R1 part and embedded separately. Five-µm-thick paraffin sections were then mounted on Superfrost Plus microscope slides.

#### Immunohistochemistry of sciatic nerve: myelin basic protein (MBP) and Neurofilament (NF)

After the deparaffinization/rehydration from xylene to distilled water, antigen retrieval was obtained in 0.1% Trypsin (T7168, Sigma-Aldrich, Merck, Darmstadt, Germany) for 30 min at 37 °C. Sections were then blocked with 5% bovine serum albumine in PBS-2.5% Triton X-100 (X100, Sigma-Aldrich) for 30 min. After washing in PBS-T (phosphate buffered saline with Tween 20), sections were incubated with the primary antibody for Myelin Basic Protein (MBP, Rat IgG2b, MCA409S BioRad, Puchheim, Germany, dilution 1/100) mixed with the primary antibody for Neurofilament-200 (NF200, Chicken IgY, ab72996, Abcam, Cambridge, UK), dilution 1/1000 in 1.5% BSA in PBS-T overnight at 4 °C. After washing in PBS-T, secondary antibodies AlexaFluor™488-conjugated Donkey anti-Chicken IgY (703546155 Jackson Immunoresearch, Cambridge, UK) and AlexaFluor™568-conjugated Goat anti-Rat IgG (A11077, Life Technologies, Thermo Fisher Scientific, Waltham, MA USA), both diluted at 1/500 and mixed together were incubated for 30 min at room temperature. After washing in PBS, sections were counterstained with 0.5 µg/mL DAPI (Roche-10236276001, Sigma-Aldrich) for 10 min. Slides were mounted in Mowiol 4–88 prepared according to manufacturer’s recommendations (475904, Merck Millipore, Basel, Switzerland).

#### Quantification of myelin and axons

Quantification of myelin content and axon areas was performed using the HALO image analysis software (Version 2.3, Indica Labs, Corrales, NM, USA). The regions of the nerves to be analyzed were outlined manually. Nerve fiber bundle regions were subsequently detected using a random forest classifier (Tissue Classifier available in HALO, version 2.3). This classifier has been trained on a small random subset of images (approximately 5%), with 3 classes for “tissue”, “background” and “other” (general class for preparation-related image artifacts).

Myelin content and axon areas were analyzed in the classified regions using the “CytoNuclear FL v1.4” algorithm (Indica Labs). Following detection of an individual axon, its outline was expanded by a given distance of 2 µm, forming a ring around the axon as the region of interest to assess the myelin content. In case axons were closer to each other than two times the distance, the ring area was reduced accordingly. In the myelin region of interest, the sum of the fluorescent signal intensity has been computed, resulting in the total myelin content. The area of the myelin region of interest has been used for normalization of the fluorescent intensity, resulting in an estimate of the mean myelin content per axon.

For axons, the stained positive area and the area of the nerve tissue bundles were measured. As readout the sum of the axon areas was normalized to the bundle area. The values are given in percent. In addition, the mean area of axons was computed, and the values are provided in µm^2^.

#### Immunohistochemistry for muscle fiber typing and central nuclei

Frozen muscle specimens were transferred to a −20 °C cryostat (Leica CM3050S; Leica Microsystems, Wetzlar, Germany) on dry ice. Each specimen was cut transversely and sections were collected at the midpoint of the muscle. The cryosections were then rehydrated in PBS and used for multiple immunostainings, combining mouse monoclonal primary antibodies anti-myosins (Developmental Studies Hybridoma Bank, University of Iowa, Iowa City, IA, USA) with rabbit polyclonal antibody anti-laminin (L9393, Sigma-Aldrich): mouse monoclonal primary antibody anti-myosin 7 (*myhc7* for Myosin Heavy Chain-1, MYHC-1, positive fibers) clone BA-D5 IgG2b (DHSB ACE21886), mouse monoclonal primary antibodies anti-myosin 4 (*myhc4* for MYHC-2b fibers) clone BF-F3 IgM (DSHB ACE28767) and anti-myosin 2 (*myhc2* for MYHC-2a fibers) clone SC-71 IgG1 (DHSB ACE28766). Cryosections were incubated for 2 hours with all primary antibodies at the concentration of 5–7 μg/mL except for the clone BF-F3 used at the concentration of 15 μg/mL. After washing, the slides were incubated with the following secondary antibodies (all from Life Technologies, Thermo Fisher Scientific), diluted 1:500, with a final working concentration of 0.4 μg/mL: AlexaFluor™488-conjugated goat anti-rabbit IgG (A-11070), AlexaFluor™555-conjugated goat anti-mouse Ig2b (A-21147), AlexaFluor™555-conjugated goat anti-mouse IgG1 (A-21120) and AlexaFluor™633-conjugated goat anti-mouse IgM (A-21426). All antibody incubations were carried out in PBS containing 0.5% triton X-100 (X100, Sigma-Aldrich) (PBST), 2% normal goat serum (G6767, Sigma-Aldrich), and proteases inhibitor cocktail (Roche-11836170001, Sigma-Aldrich), and washes were carried out with PBST. Subsequently, sections were counterstained with 0.5 µg/mL DAPI (10236276001, Roche) for 10 min. After washing, sections were post-fixed with 1% ParaFormaldehyde for 10 minutes. Slides were mounted in Mowiol 4–88 prepared according to manufacturer’s recommendations (475904, Merck Millipore). All the staining steps were done at room temperature. Slides were stored at 4 °C for imaging and analysis.

#### Imaging and quantification of fiber types

Histology slides were scanned using an Olympus VS120 scanner (Olympus, Volketswil, Switzerland) at 20x magnification. For quantification of the proportion of each fiber type on a given muscle section, the proprietary in-house image analysis platform ASTORIA (Automated Stored Image Analysis, Novartis Pharma AG, Basel, Switzerland), based on MS Visual Studio 2010 and Matrox MIL V9 libraries (Matrox Inc, Quebec, Canada), was used. Myofiber type, numbers and CSA were evaluated after segmenting sections based on laminin staining. Central nuclei were detected and counted after segmenting sections based on laminin and DAPI staining.

For slow/fast myofiber segmentation and area quantification, the following steps were performed: ROIs were manually specified for image segmentation from the slow/fast myosin/laminin immunostained slide image; oblique and transverse cut regions were excluded. Tile images from each ROI were then created at magnification 5x and analyzed by the algorithm. Analysis consisted of the detection of myofiber boundaries based on laminin staining, extraction of myosin staining to characterize cells, subsequent post-processing (segmentation, merging), and finally classification of each myofiber as either slow or fast. Measured parameters comprised the number of slow and fast myofibers, and additional morphometric parameters characterizing fiber shape.

### Gene expression

Lateral gastrocnemius muscle samples were lysed in 1 ml of TRIzol reagent (15596018, Thermo Fisher Scientific) respectively in Lysing Matrix D or Lysing Matrix A tubes (116913500, MP Biomedicals, Illkirch-Graffenstaden, France). Total RNA was isolated with TRIzol reagent according to the manufacturer’s instructions and TURBO DNA-free™ Kit (AM1907, Thermo Fisher Scientific) was used for complete DNA digestion. RNA (500 ng) was then reverse transcribed to cDNA using the High-Capacity RNA-to-cDNA™ Kit (4387406, Thermo Fisher Scientific).

Quantitative polymerase chain reaction (qPCR) was carried out using 10 ng of cDNA per each sample and TaqMan® Universal PCR Master Mix (4324018, Thermo Fisher Scientific). Gene-specific primers with FAM-labeled probes were from Thermo Fisher Scientific: *Chrna1*, Mm00431629_m1; *Mafbx*, Mm00499518_m1; *Murf1*, Mm01185221_m1; *Myhc1*, Mm01332489_m1; *Myhc2*, Mm00454991_m1; *Myhc4*, Mm01332541_m1; *Myhc7*, Mm00600555_m1; *Myog*, Mm00446194_m1. *Gapdh*, Mm99999915_g1; *Tbp*, Mm01277042_m1; *Hprt*, Mm01545399_m1; and *Rplp0*, Mm00725448_s1 were used as internal control.

PCR cycling conditions on software supplied with ViiA™ 7 System (Applied Biosystems, Foster City, CA) were as follows: 50 °C for 2 min, 95 °C for 10 min, 40 cycles at 95 °C for 15 s, and 60 °C for 1 min. Data were expressed as Ct values and used for the relative quantification of targets with the ΔΔCt calculation to give N-fold differences. Data were transformed through the equation 2^−ΔΔCt^.

### Statistics

Prism 7 (GraphPad Software, La Jolla, CA) was used for statistical analysis: gene expression, histological, MRI and electrophysiological data were analyzed using multiple unpaired two-tailed Student’s t test; regional MTR data were analyzed with two-way ANOVA with Dunnett’s multiple comparisons test. Non-parametric Mann-Whitney tests were used to analyze toe spreading data. Statistical significance was considered at p ≤ 0.05.

## Supplementary information

Supplementary figures

## Data Availability

The datasets generated and/or analysed during the current study are not publicly available due to internal regulations from Novartis on data availablity. Data may however become available from the corresponding author upon reasonable request and pending approval by Novartis.
