# Supplementary material for: Magnetic Resonance Imaging as a Biomarker in Rodent Peripheral Nerve Injury Models Reveals an Age-Related Impairment of Nerve Regeneration
Source: Sci Rep. 2019 Sep 18;9:13508. doi: 10.1038/s41598-019-49850-2 (PMC6751200; doi:10.1038/s41598-019-49850-2)
Supplement: Supplementary file 1 — Supplementary figures [file 41598_2019_49850_MOESM1_ESM.pdf]

Magnetic Resonance Imaging as a Biomarker in Rodent Peripheral Nerve Injury Models Reveals an Age-Related Impairment of Nerve Regeneration

Elisa Giorgetti, Michael Obrecht, Marie Ronco, Moh Panesar, Christian Lambert,

Nathalie Accart, Arno Doelemeyer, Mark Nash, Michael Bidinosti, Nicolau Beckmann*

Novartis Institutes for BioMedical Research, Musculoskeletal Diseases Department

CH-4056 Basel, Switzerland

*Correspondence: PD Dr. Nicolau Beckmann
Novartis Institutes for BioMedical Research
Musculoskeletal Diseases Department
Fabrikstr. 28.3.04
CH-4056 Basel – Switzerland
e-mail: nicolau.beckmann@novartis.com

SUPPLEMENTARY FIGURES

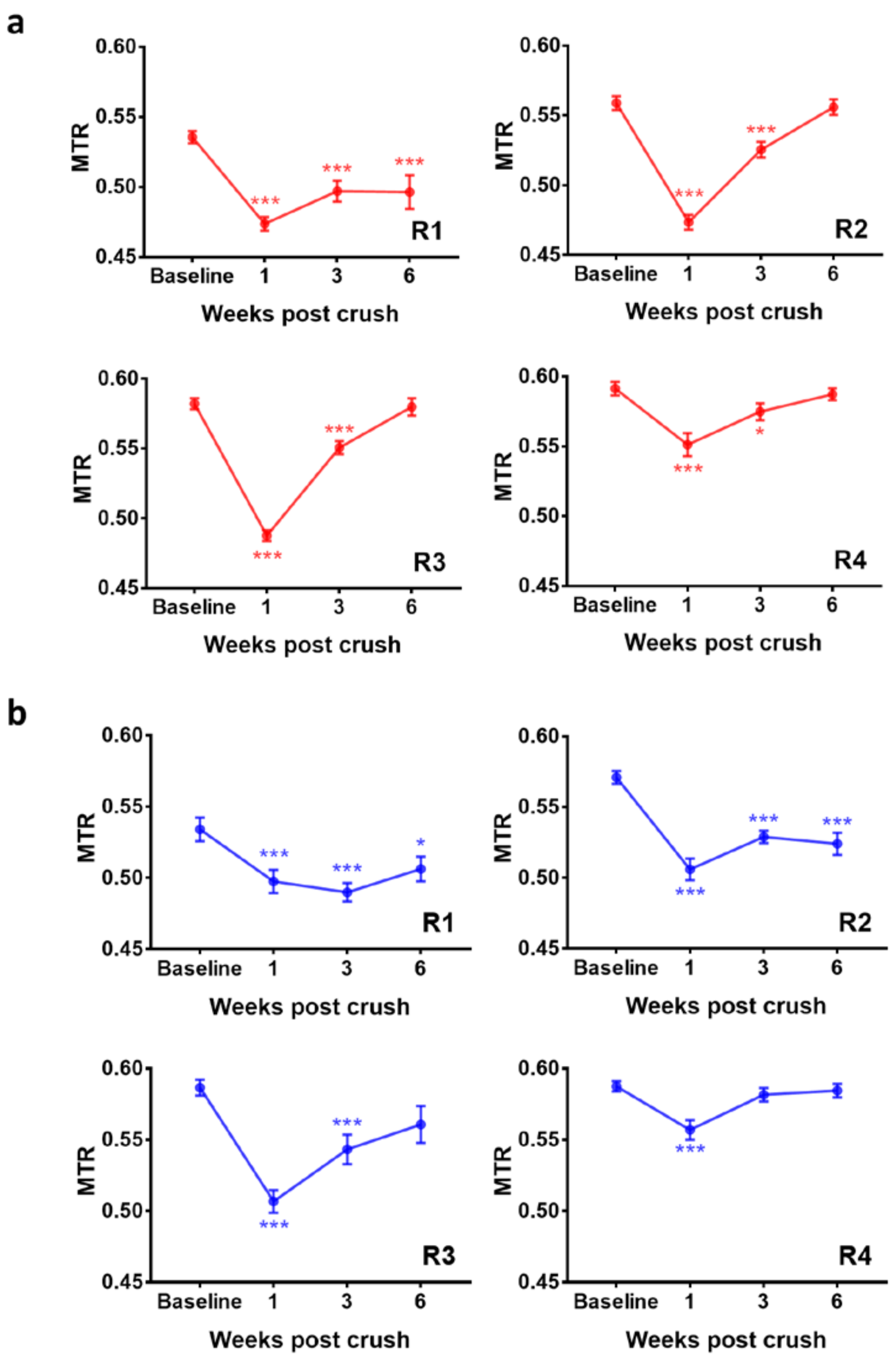

Suppl. Figure 1. Regional MTR analyzed at baseline and at weeks 1 and 6 after SNC in young **(a)** and old **(b)** mice (Means ± SEM; *$0.01 < p < 0.05$, ***$p < 0.001$, comparisons to baseline values for each region). Number of animals in each age group: n=18 SNC at baseline and week 1, n=12 SNC at week 3, and n=6 SNC at week 6.

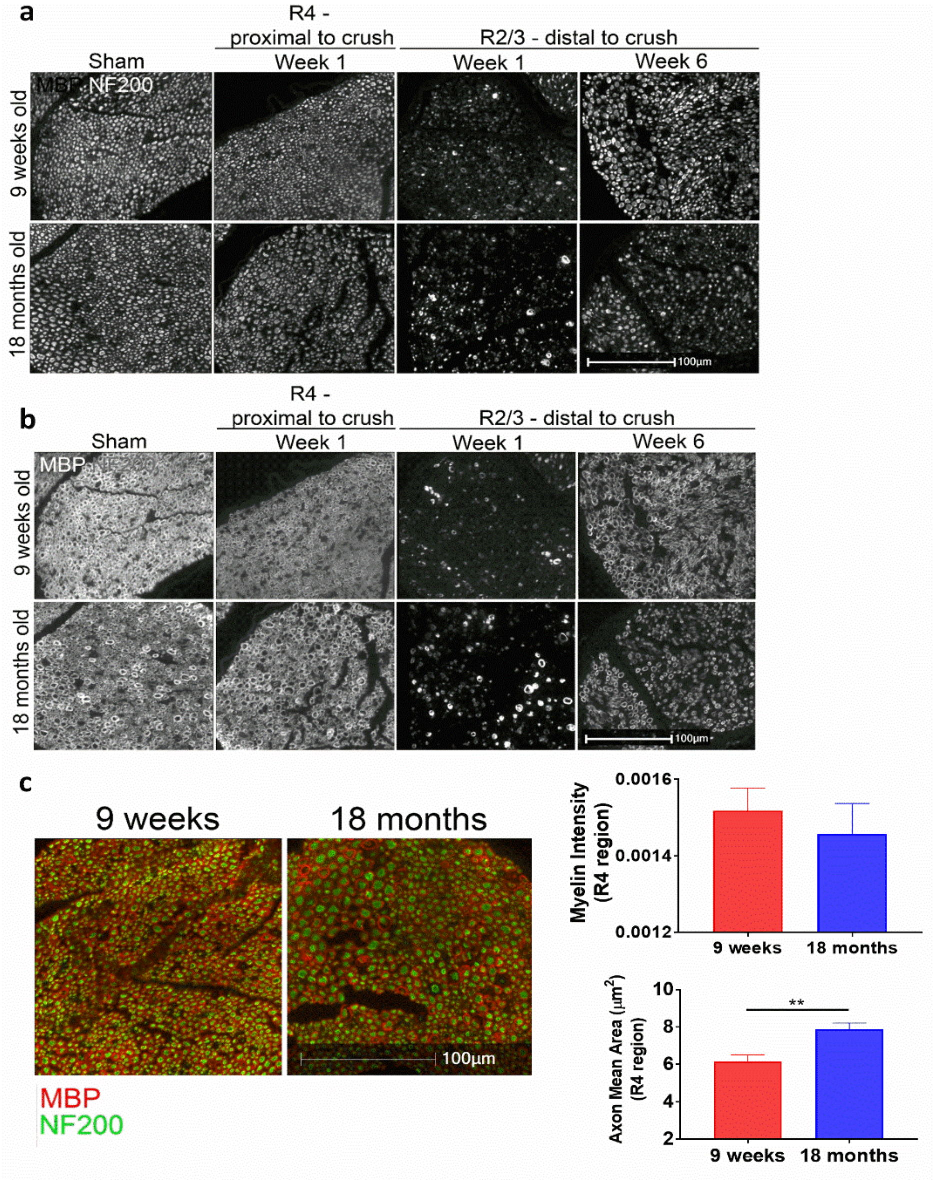

Suppl. Figure 2. Histology of sciatic nerves from young and old mice. **(a)** Representative images of sciatic nerve transections stained with anti-NF200 (axons) antibodies in regions proximal (R4, at week 1 after SNC) and distal to the crush site (R2/3, at week 1 and 6 after SNC). Proximal region R4 of sham-operated nerves has been included for comparison. **(b)** Representative images of nerve transections stained with anti-MBP (myelin) antibodies in regions proximal (R4, at week 1 after SNC) and distal to the crush site (R2/3, at week 1 and 6 after SNC). Proximal region R4 of sham-operated nerves has been included for comparison. **(c)** Representative confocal images of sciatic nerve transections stained with anti-MBP and anti-NF200 antibodies with corresponding quantification of myelin intensity and axonal mean area (µm^2) in region R4 (Means ± SEM; **$p < 0.01$, n=9 mice).

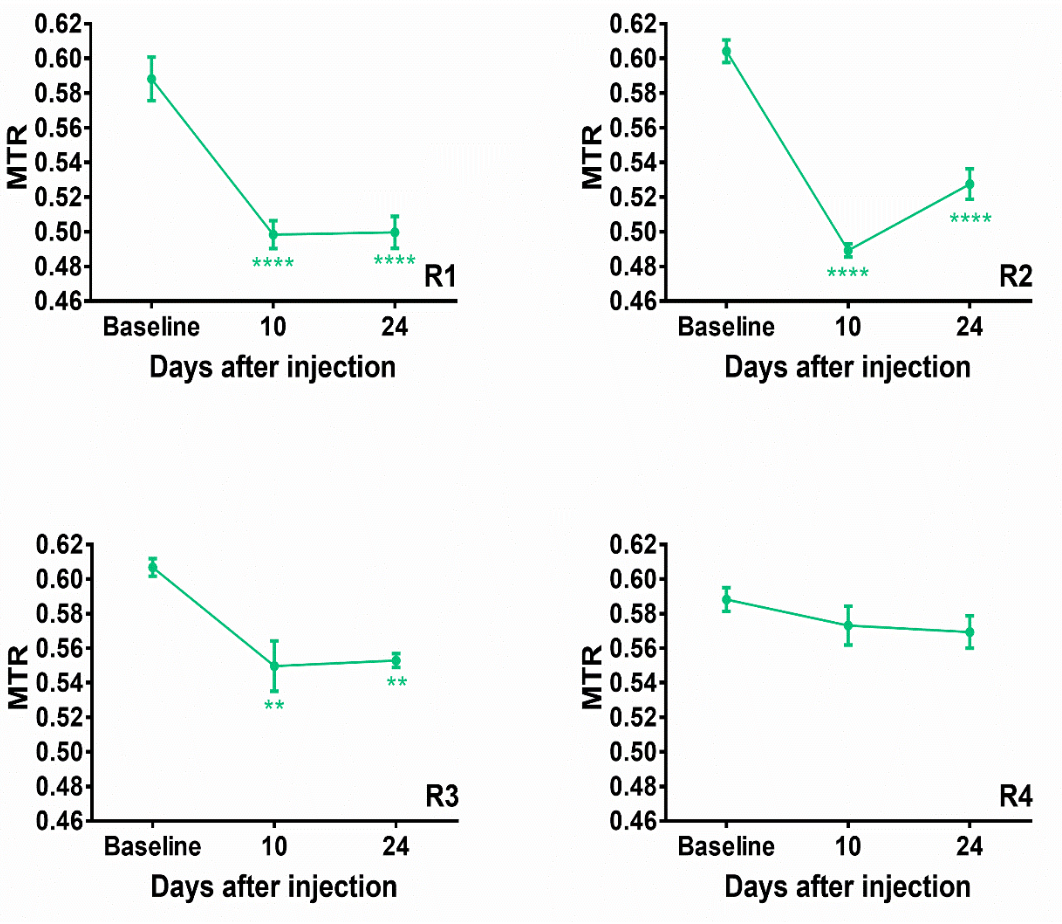

Suppl. Figure 3. Regional MTR analyzed at baseline and at day 10 and 24 after LCP injection in young rats and expressed in absolute values (Mean ± SEM; **p< 0.01, ****p<0.0001, comparisons to baseline values for each region). Number of rats: n=6 LCP for each age group.
